# Two cold-sensitive neurons within one sensillum code for different parameters of the thermal environment in the ant *Camponotus rufipes*

**DOI:** 10.3389/fnbeh.2015.00240

**Published:** 2015-09-03

**Authors:** Manuel Nagel, Christoph J. Kleineidam

**Affiliations:** Behavioral Neurobiology, Department of Biology, University of KonstanzKonstanz, Germany

**Keywords:** sensillum coelocapitulum, temperature sensing, brood care, sensory physiology, single sensillum recording, auriga FIB-FESEM, social insects

## Abstract

Ants show high sensitivity when responding to minute temperature changes and are able to track preferred temperatures with amazing precision. As social insects, they have to detect and cope with thermal fluctuations not only for their individual benefit but also for the developmental benefit of the colony and its brood. In this study we investigate the sensory basis for the fine-tuned, temperature guided behaviors found in ants, specifically what information about their thermal environment they can assess. We describe the dose-response curves of two cold-sensitive neurons, associated with the sensillum coelocapitulum on the antenna of the carpenter ant *Camponotus rufipes*.One cold-sensitive neuron codes for temperature changes, thus functioning as a thermal flux-detector. Neurons of such type continuously provide the ant with information about temperature transients (TT-neuron). The TT-neurons are able to resolve a relative change of 37% in stimulus intensity (ΔT) and antennal scanning of the thermal environment may aid the ant’s ability to use temperature differences for orientation.The second cold-sensitive neuron in the S. coelocapitulum responds to temperature only within a narrow temperature range. A temperature difference of 1.6°C can be resolved by this neuron type. Since the working range matches the preferred temperature range for brood care of *Camponotus rufipes*, we hypothesize that this temperature sensor can function as a thermal switch to trigger brood care behavior, based on absolute (steady state) temperature.

## Introduction

Insects are small animals and consequently, their body temperature is close to environmental conditions. Since environmental temperature conditions have a crucial impact on individual fitness, many different strategies evolved to cope with temperature fluctuations and temperature regulation. Adaptations for temperature regulation in insects comprise physiological and/or behavioral responses in order to increase or decrease body temperature (Heinrich, 1996). One example of thermoregulatory behavior is warming-up in flying insects at suboptimal temperature conditions. Flying insects require high thoracic temperature for proper function of their flight muscles. Whereas some species (e.g., bees, dragonflies) increase thoracic temperature by shivering and hence producing heat actively with their flight muscles before lifting up, other species (e.g., butterflies, dragonflies) select temperature conditions for passive temperature increase by sunbasking (Heinrich, 1974, 1975; Heinrich and Casey, 1978). Superoptimal temperature conditions, e.g., high temperatures can cause serious impairments or even become lethal (reviewed in Neven, 2000). Insects can cool down by seeking cooler areas, by increasing evaporative cooling during flight and by adjusting the flow of the haemolymph (Heinrich, 1975; Heinrich and Casey, 1978; Prange, 1996; Roberts and Harrison, 1998).

In social insects, thermoregulatory behaviors evolved that allow controlling temperature inside their hives or nests. Such nest thermoregulation allows social insects to successfully cope with thermal fluctuations for their direct individual benefit and to provide favorable conditions for the development of their virtually immobile brood. Ultimately, collective thermal homeostasis promotes colony growth and inclusive fitness (Porter and Tschinkel, 1993). Social insects with wings, like bees and wasps, use their flight apparatus to control temperature. They can incubate the brood by heating up their thoracic temperature or start fanning to decrease temperature in their hives through evaporative cooling (Heinrich, 1981). Wingless social insects like ants control nest temperature passively by isolation and absorption of solar radiation at the nest site, and actively by sun basking individuals that transfer heat from outside to the nest interior (Goesswald and Kneitz, 1964; Kneitz, 1966). Elaborate nest structures with sophisticated ventilation properties and many different nest chambers provide diverse microclimatic conditions. Ants translocate their brood to chambers with favorable microclimatic conditions (Roces and Núñez, 1989; Roces, 1995; Weidenmüller et al., 2009; reviewed in Jones and Oldroyd, 2006). During this brood care behavior, ants show remarkable sensitivity and precision in detecting the preferred temperature.

In the carpenter ant *Camponotus rufipes*, workers prefer temperatures around 30°C (Weidenmüller et al., 2009). Throughout the day, temperature preferences change, and the ants deposit brood at 28°C in the early morning and at 32°C in the evening. Additionally, the individual response threshold for brood translocation depends on the ant’s thermal experience during development and as adults (Weidenmüller et al., 2009). It is unknown, which sensilla and their associated sensory neurons provide the information, necessary for such fast and fine-tuned behaviors.

The most prominent sensory organ in insects is the antenna that is equipped with different types of sensilla. Most thermo-sensitive neurons in insects described so far are associated with peg-in-pit sensilla. The common Bauplan of these sensilla consists of a cuticular pit containing a sensory peg. Three different types of peg-in-pit sensilla are described in ants: the S. ampullaceum, the S. coeloconicum, and the S. coelocapitulum (Yokohari, 1983; Kleineidam and Tautz, 1996; Kleineidam et al., 2000; Nakanishi et al., 2009; Ruchty et al., 2009). The cuticular pits of the S. ampullaceum and S. coeloconicum are connected to the environment only by a small aperture (Kleineidam et al., 2000; Ruchty et al., 2009). This suggests an adaptive function of the peg-in-pit structure like shielding the sensory neurons from turbulences in the environment.

A variety of thermo-sensitive neurons are described for the S. coeloconicum and S. ampullaceum in insects, either combined with chemo-sensitive neurons (Lacher, 1964; Altner, 1977; Davis, 1977; Altner et al., 1981; Kleineidam and Tautz, 1996) or forming a sensory triad with two hygro-sensitive neurons (Waldow, 1970; Tichy, 1979; Altner et al., 1981; Yokohari et al., 1982; Altner and Loftus, 1985; Piersanti et al., 2011). In ants, thermo-sensitive neurons are known to be associated with the S. coeloconicum and S. ampullaceum (Kleineidam and Tautz, 1996), and one phasic-tonic cold-sensitive neuron associated with the S. coeloconicum was described in great detail (Ruchty et al., 2009, 2010a,b). This cold-sensitive neuron is highly sensitive for transient temperatures and adapts to steady state temperature. The adaptation property increases the temperature range in which thermal changes can be detected, but information about steady state temperature is not encoded (Ruchty et al., 2010b). In contrast to the above described coeloconic and ampullaceal sensilla, the S. coelocapitulum is characterized by a mushroom like protrusion of the sensory peg at the antennal surface within a less prominent cuticular pit, compared to the S. coeloconicum or S. ampullaceum (Dietz and Humphrey, 1971; Esslen and Kaissling, 1976; Yokohari et al., 1982; Yokohari, 1983; Nakanishi et al., 2009; Tichy and Kallina, 2014). In the honey bee, one of the associated receptor neurons of the S. coelocapitulum has been identified as being thermo-sensitive but this neuron was not further characterized (Yokohari, 1983). In ants, the function of the S. coelocapitulum and the physiological properties of the associated sensory neurons are unknown. Based on the previous report of Yokohari (1983), we hypothesize that the S. coelocapitulum in ants also contains at least one thermo-sensitive neuron.

In the present study, we investigated the S. coelocapitulum in the carpenter ant *Camponotus rufipes*, using extracellular recordings (single sensillum recording). The single sensillum recordings were performed during fast and slow gradual temperature changes. The neuronal activity profiles of two cold-sensitive sensory neurons are described by distinct dose-response curves, showing parallel parameter extraction within one sensory modality (temperature). The different temperature parameters extracted by each type of neuron are discussed in the context of the elaborate temperature guided behavior in ants.

## Materials and Methods

### Animals and Preparation

Workers were obtained from a mature colony of *Camponotus rufipes*. The colony was collected in 2011 from La Coronilla, Uruguay, by Oliver Geißler and kindly provided by F. Roces (University of Würzburg). The colony was kept at 25°C and 50% rH at the University of Konstanz with a 12 h:12 h L/D-cycle. Honeywater and cockroaches or locusts were provided twice a week. Workers were collected from the colony and immobilized on a glasslide with adhesive tape. The mandibles and the scape were mounted in dental wax (Surgident, Heraeus Kulzer, Germany). The flagellum of the antenna was mounted under visual control (Leica S8AP0, Leica, Microsystems, Wetzlar, Germany) with water based white-out correction fluid (Tipp-Ex, Bic, France) exposing the lateral-ventral side upwards.

### Morphological Investigation by SEM, FIB-FESEM, cLSM, Light Microscopy and TEM

The correct identification under light microscopic conditions, as used for the single sensilla recordings was confirmed by using scanning electron microscopy (SEM) and confocal laser scanning microscopy (cLSM). The morphological characteristics described by SEM-Images were used to identify the S. coelocapitulum in preparations investigated by cLSM. Specimens in which S. coelocapitula were identified by cLSM were subsequently investigated under light microscopic conditions. The peg-in-pit morphology of the S. coelocapitulum causes specific light refraction that allows the identification on the antennae of live animals for electrophysiological recordings.

The microscopic investigations were done with bisected antennal tips. For bisection, the animals were fixed as described above, and the tip of the antenna was covered with wax. The embedded antennal tips were then cut with a broken razorblade parallel to the glass slide. After bisecting the distal segments, the two halves were transferred in 1M KOH for 15 min and then washed in 70% ethanol (2 times, 5 min each) and cleansed in an ultrasonic bath for 2 min. The tip-cuts were washed and dehydrated in 100% ethanol (two times, 10 min).

For SEM (*n* = 21 (sensilla)) and focus ion beam (FIB-FESEM, *n* = 2) investigations the antennal tip-cuts (*n* = 4) were gold sputtered (Sputtercoater SCD 030, Balzers, Germany). The SEM-images of the antennal surface and the FIB-FESEM-investigations were taken at a resolution of 1024 × 768 pixel with a SE2-detector at the AURIGA-system (Zeiss, Jena, Germany).

For cLSM investigations, the dehydrated tip-cuts were embedded in DPX-Mountant for histology (No. 44581, Sigma-Aldrich, Germany). The embedded tip cuts were investigated with cLSM (LSM 510 Meta system Zeiss, Germany) using a 63×/1.4 oil objective and an Argon/Neon-laser with 488 nm. The image stacks were analyzed, using AMIRA 5.2 (Mercury Computer Systems, Berlin, Germany) and a projection view of the antennal tip allowed the identification of the S. coelocapitulum. We used a transmission light microscope (Examiner.A1, Zeiss, Germany) and a 500 fold magnification (LD EC Epiplan-Neofluar 50×/0.55 DIC, Zeiss, Germany) for light microscopic investigation and visual control during extracellular recordings.

### TEM Procedure

The fine-structure of the S. coelocapitulum (*n* = 2) was investigated using transmission electron microscopy (TEM). Identification of the S. coelocapitulum under TEM conditions was achieved using cLSM prior to the ultrathin sections for TEM. For the TEM preparation, the two distal segments of the antenna were cut with a razor blade, transferred in 0.1 M Na-cacodylat-saline with 2.5% glutaraldehyde and 2.5% glucose and incubated over night at 4°C. The prepared segments were rinsed in 0.1M Na-cacodylat-saline (3 times, 10 min), transferred into Na-cacodylat-solution with 1% osmiumtetroxid and incubated at 4°C for 12 h. The segments were again rinsed in Na-cacodylat-solution (3 times, 10 min), dehydrated in a graded ethanol series (30%, 50%, 70%, 80%, 90%, 100%), and embedded in Spurr-Epon-Araldit. The embedded segments were cut into 5 μm thick sections, placed on a glass slide and covered with a cover slide. The sections were investigated under cLSM conditions as described for the tip-cuts. Subsequent to a successful identification of S. coelocapitula in single sections, those sections were cut in sections of 850 nm and investigated with a TEM (Omega 912, Zeis, Germany).

### Temperature Stimulation Set-Up

Temperature stimulation was achieved with a temperature-controlled airstream of 1 l/min. Filtered and dried air (4–7% rH at 25°C) was split into two separate airstreams. The two separate airstreams were controlled by proportional flow meters (SLPM 35831, Analyt-MTC, Germany), using instrument control of LabView (LabView 2011, National Intstruments, USA). The airstreams were cooled down or heated, respectively, with a waterbath-based counter flow heat exchange system (workshop built, University of Konstanz). After cooling/heating, the two separate airstreams were fused close to the recording side. The ratio of the mixture allowed the application of different temperatures and different temperature changing rates at a constant flowrate. At the beginning of each stimulation, the steady state temperature was set to 25°C, matching the rearing temperature of the animals and experiments were conducted at room temperature between 24°C and 27°C.

The temperature stimulation protocol started either with a cold- or a heat-stimulus at a given temperature changing rate to a plateau phase of steady state temperature for 500 s followed by a temperature increase or decrease, respectively, back to 25°C. The same temperature changing rate was applied for the two temperature transition-phases, but with different arithmetic signs. The temperature changing rates in the first second after stimulus onset ranged from −0.43°C/s to +0.43°C/s, and the smallest temperature changing rate we used was 0.01°C/s. We calculated the actual temperature, using the average measure in 1 s windows, and in order to compensate for noise, we applied a Gauss-filter to the temperature measurements. In order to achieve temperature recordings that correspond to the stimulus intensities, the thermocouple was placed right behind the recording site.

### Recording of Sensory Neuron Activity and Temperature Stimulus

The neuronal activity of the sensory neurons associated with the S. coelocapitulum was investigated by extracellular recordings. The precise positioning of the reference and the recording electrode was controlled by two digital micromanipulators (NanoControlNC40, Kleindiek, Germany). As reference electrode, an electrolytically sharpened tungsten electrode was inserted deep into the last segment of the flagellum. As recording electrode, a glass capillary (1B100F-3, Precision Instruments, USA) was used and two electrodes were produced by a micropipette-puller (P-97 Flaming/Brown, Sutter Instrument, USA). The recording electrodes had a resistance of 7–60 MΩ when filled with 1M KCl. The recordings were band passed filtered (100 Hz–3 kHz) and amplified 1000× (BA-03X amplifier, npi, Germany). Additionally, a digital filter (Humbug, Quest Scientific, Canada) was used to reduce the electrical noise. The voltage signals of the sensory neurons were sampled at a frequency of 25 kHz.

Temperature was recorded with a PFA-insulated T/C-thermocouple (5TC-TT-TITI, Omega, US and Canada), positioned 0.2–0.5 cm behind the recording side. The thermocouples were connected to a Thermocouple input module (NI9214, National Instruments, USA) at a sampling rate of 4 Hz in high-resolution mode. This configuration resulted in an accuracy of temperature measures of 0.01°C. The voltage of the neuronal signals and temperature were recorded simultaneously.

The neuronal activity of the sensory neurons was recorded by placing the electrode in close proximity to a S. coelocapitulum. The temperature-controlled airstream was directed onto the antennal tip along the longitudinal axis of the antenna. The recording electrode was inserted in an off-axis angle into the shallow depression next to the mushroom like protrusion of the S. coelocapitulum. This arrangement of the recording electrode with respect to the airstream ensures minimal impact of the electrode to the stimulus application, and the very sharp tip of the electrode may only have a small impact on additional heat-flow from the electrode to the sensillum or* vice versa*. The stereotyped positioning of the electrode may be one reason why we measured in most cases only two of the probably three neurons of the S. coelocapitulum (Nakanishi et al., 2009).

### Data Analyses and Statistics

The extracellular recordings with sharp glass electrodes allowed measuring small voltage changes (spikes), corresponding to action potentials (APs) of the neurons. The amplitude and the shape of the spikes depend on physical properties of the sensillum, size of the neurons and also the vicinity of the recording electrode to the sensory neurons. The spikes of different sensory neurons can be discriminated based on shape and amplitude, and thereby activity can be assigned to different sensory neurons.

In those cases where more than one sensory neuron was recorded, the signal-to-noise ratio was good for one of the sensory neurons, whereas the spike-amplitude of a second neuron was close to noise level and segregated from noise by the shape of its spikes. The spikes were sorted using Spike2-software (Spike2 v7.03, Cambridge Electronic Design, UK) and further analyses were done using R-software (RDC-Team, 2012). If not otherwise stated, all calculations on the temperature stimulus and the neuronal activity were done in 1 s windows.

#### Analysis of a Cold-Sensitive Neuron, Coding for Temperature Changes (TT-Neuron)

In all recordings, the spikes with high amplitude can be assigned to a cold-sensitive neuron, responding to temperature changing rates (TT-neuron), and adaptations during steady state temperature conditions. We quantified the response properties using the instantaneous frequency (IF; reciprocal of the inter spike interval) for describing the neuronal response and the temperature changing rate as the adequate stimulus.

Even without temperature stimulation, the IF of the TT-neurons is variable, and in order to compensate for this variability, we calculated the median IF in a 1 s bin. The average of the activity at steady state condition (10 s before stimulus onset) was used to normalize the neuronal activity during one recording and resulted in a normalized instantaneous frequency (nIF in %). This normalization of the median IF allows the comparison of different TT-neurons across animals. We averaged the nIF 10 s before the stimulus onset and calculated the corresponding SD. The two fold SD was used as a measure of noise.

The response of the sensory neurons followed the Weber-Fechner-law, therefore we log-transformed the temperature changing rate (stimulus intensity) and described the dose-response curve, using a linear regression. We calculated a linear regression on the nIFs that were above the noise level of the resting activity during stimulation. Only measurements with a significant linear regression (*p* < 0.05) and a Pearson’s correlation index above 0.3 (moderate positive relationship) or below −0.3 (moderate negative relationship) were used for further analyses. We calculated the noise level of the nIF during temperature stimulation by calculating the two fold SD-value with respect to the linear regression.

The two parameters defining the response properties of the neuron are the smallest temperature change that elicits a neuronal response to temperature changes above noise level (detection threshold) and the slope of the regression (differential sensitivity). The differential sensitivity can further be described by the resolving power for temperature changing rates, which is calculated based on the standard deviation of each nIF to the respective linear regression (Ameismeier and Loftus, 1988).

The equation used for the calculation is

(1) $$\Delta x=\frac{\sqrt{2\sigma }}{\left|b\right|}\Phi_{\left(\text{γ}\right)}^{-1}$$

in which |*b*| is the absolute slope of the linear regression, σ^2^ the variance of nIFs with respect to the linear regression, and *γ* a required probability for detecting differences. We set the required probability to 90%, following common consensus (Ameismeier and Loftus, 1988; Zopf et al., 2014). The inverse of the distribution function (Φ^−1^_(0.9)_) of a standardized, normally distributed, random variable results in a value of Φ^−1^_(0.9)_ = 1.28 (adapted from Geigy et al., 1968). The variance in the case of the linear regression is estimated by

(2) $$\sigma^{2}=\;\frac{\sum \varepsilon^{2}}{n-1}$$

where *ε* is the deviation for each nIF to the linear regression. The degree of freedom for linear regressions is calculated by the number of curves *I* and the number of measurements *n*.

#### Analysis of a Cold-Sensitive Neuron, Coding Temperature Only Around 30°C (ST-Neuron)

A second cold-sensitive neuron with smaller spike-amplitudes can be identified during gradual temperature increase. The neuronal activity changed in a very limited temperature range (working range). As it was not possible to test different steady state temperatures in this narrow working range, we tested the neurons response following very slow temperature increases, close to steady state temperature conditions. Based on our conclusions drawn from the neuron’s properties, we refer to it as steady temperature neuron (ST-neuron) in the following.

Since the ST-neuron lacked a phasic response in our measurements, we quantified the neuronal response by the mean rate of the neuron in 1 s windows. The mean rate was stable around starting conditions (25°C) and decreased with increasing temperature until it reached a significantly lower activity level at higher temperatures. A sigmoidal regression model based on a Gauss-Newton iteration was applied on the mean rate of the ST-neurons (Sarkar and Andrews, 2013). The slope of the regression describes the neuron’s sensitivity. We described the neuron’s temperature working range by its neuronal activity that is 10% lower than the high activity level at low temperatures and 10% higher than the low activity level at high temperatures. We assessed the resolving power of ST-neurons for steady state temperature with the formula for linear regression (equation I). A close approximation was done using a linear regression on the measurements in a temperature range of ±1°C around the turning point (highest sensitivity).

## Results

### External and Internal Morphology and Location of the S. coelocapitulum

**Figure 1:**
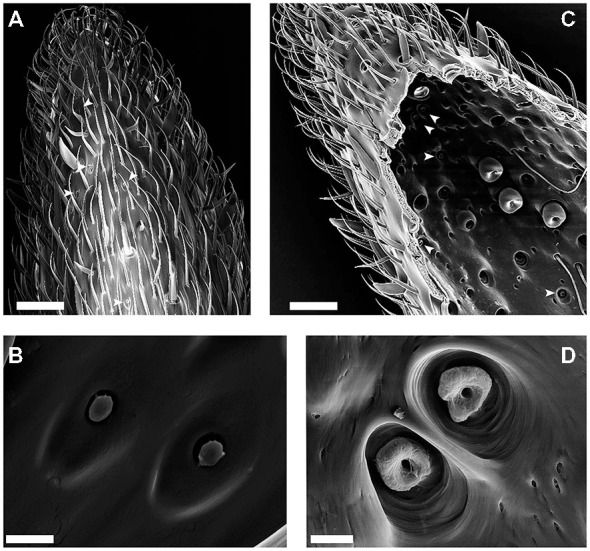
**Scanningelectron microscopy (SEM)-images of the external and internal cuticular morphology of the S. coelocapitulum. (A)** Overview of the distal part of the ventral-lateral side of the 10th antennal segment. The external morphology of the S. coelocapitulum is characterized by an oval depression with a central, mushroom-like protrusion (arrowheads). **(B)** Close-up of a pair of S. coelocapitula. **(C,D)** Images of the internal side of the antennal cuticle after tissue removal. **(C)** Overview of the internal cuticular structures of the distal end of the antennal tip. The prominent pits of the S. coeloconica are visible as a cluster on the ventral-lateral side of the antenna. The smaller cuticular pits (arrowheads) are the S. coelocapitula. **(D)** Close-up of the base of the pit and peg with an irregular, donut-like surface. Scale in **(A,C)**: 20 μm. Scale in **(B,D)**: 2 μm.

The S. coelocapitula are clustered at the most distal antennal segment of the flagellum. In *C. rufipes* workers, about 10–12 S. coelocapitula are located ventral-lateral and more distal than the cluster of S. coeloconica and S. ampullacea (Figures 1A,C). These two clusters of peg-in-pit sensilla do not overlap.

The overall morphological characteristics comprise a mushroom-like protrusion centrally in a shallow, oval depression on the antennal cuticle (Figures 1A,B). We investigated the external morphology of the S. coelocapitula (*n* = 15) in ten antennae obtained from six different individuals. The average length of the oval depression measures 5.47 μm (SD = 0.25 μm, *n* = 15) and the width 3.07 μm (SD = 0.27 μm, *n* = 15). The surface of the central protrusion is of irregular texture and corresponds to the tip of the sensory peg. On average, the protrusion is 1.25 μm (SD = 0.07 μm, *n* = 15) long and 0.90 μm (SD = 0.06 μm, *n* = 15) wide. At the antennal surface, the blunt peg is surrounded by a cleft of 0.29 μm in width (SD = 0.08 μm, *n* = 15, Figure 1B).

Often, two S. coelocapitula appear in close vicinity on the antennal surface (Figure 1B). This typical arrangement of the two sensilla and the classification as peg-in-pit sensilla (Yokohari et al., 1982; Nakanishi et al., 2009) allows the identification of the pit structures at the internal cuticular surface (Figure 1D). We used the most prominent peg-in-pit sensilla (S. coeloconica; Figure 1C) for orientation. The cuticular pits of the S. coeloconica are clearly visible (Figure 1C) and clustered on one side of the antenna. The inner surface of the pits is smooth and the base of the inserted peg can be seen as a small hole at the base of the pit. The cuticular pits of the S. coeloconica allowed the identification of the cluster of the smaller S. coelocapitula, located more distally (Figure 1C). The internal cuticular morphology of S. coelocapitula (*n* = 6) was investigated in four antennae obtained from three different individuals.

**Figure 2:**
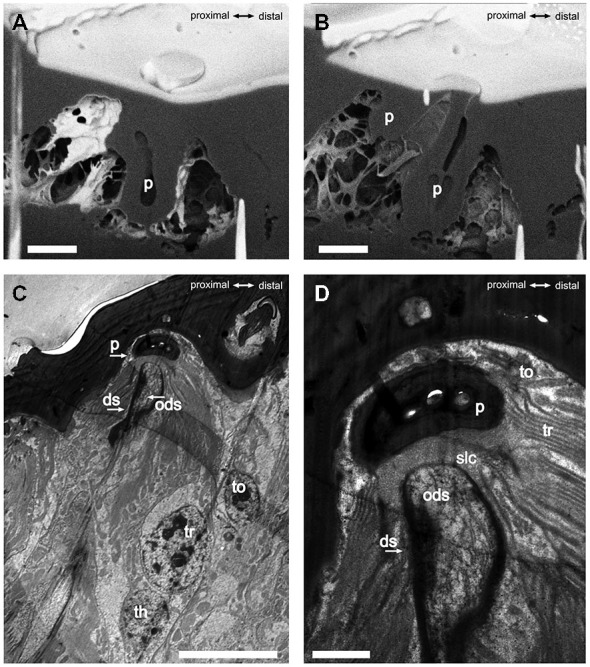
**FIB-FESEM- and TEM-images of the S. coelocapitulum. Sagittal view of the cuticular pit of one S. coelocapitulum.** The distal part of the pit is enlarged compared to the proximal part. **(B)** The compact proximal part of the pit reaches 1 μm and the hollow distal part reaches 2.5 μm deep into the antennal lumen. The insertion of the dendritic outer segments of the sensory neurons into the sensory peg is tilted. **(C,D)** Sagittal TEM-Images of two different S. coelocapitula. **(C)** The cuticular pit (p), the outer dendritic segment (ods), the dendritic sheath (ds), and three supporting cells (tormogen (to), trichogen (tr), thegocen (th)) are visible. **(D)** The lamellation of the trichogen cell and the proximal end of the pit define the sensillum lymph cavity (slc). Scale in **(A,B)**: 1 μm, scale in **(C)**: 0.5 μm, scale in **(D)**: 0.1 μm.

The S. coelocapitulum have the smallest cuticular pit of all peg-in-pit sensilla, and the pit is embedded in a dome shaped cavity within the antennal cuticle. The dome has an average length of 5.75 μm (*SD* = 0.77 μm, *n* = 6) and an average width of 4.28 μm (*SD* = 0.59 μm, *n* = 6). The cuticular inner surface of the pit is irregular, and the pit is on average 2.44 μm long (*SD* = 0.13 μm, *n* = 6) and 2.19 μm wide (*SD* = 0.19 μm, *n* = 6). The opening at the base of the peg is on average 0.51 μm (*SD* = 0.03 μm, *n* = 6). The depth of the pit and its structure was identified using the FIB-FESEM-technique. The distal part of the pit reached 2.5 μm and the proximal part only 1 μm into the antennal lumen (Figures 2A,B). Thus, the sensillum is embedded oblique in the antennal cuticle. The peg extends from the pit to the surface of the antennal cuticle, and is separated from it by a surrounding cleft (Figures 2A,B). The distal part of the pit is hollow and air filled, whereas the proximal part of the pit is reduced, and almost fused with the antennal cuticle (Figures 2A,B). The sensory peg has a cone shaped cavity and since most of the cellular tissue was removed, only remnants of the dendritic sheath remained in the cavity (Figure 2B). A dendritic sheath surrounds the outer dendritic segments of the sensory neurons, and the sensillum lymph cavity extends proximal of the pit (Figures 2C,D). Two outer supporting cells (tormogen, trichogen) surround the cuticular pit, forming the sensillum lymph cavity (Figure 2D). The inner supporting cell (thecogen) surrounds the dendritic sheath and the inner dendritic segment (Figure 2C).

**Figure 3:**
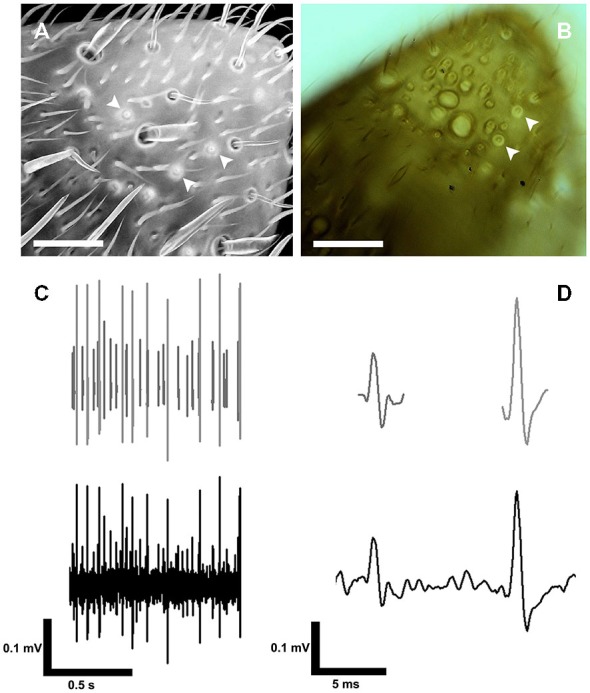
**Identification of the S. coelocapitulum and extracellular recordings of the associated sensory neurons. (A)** Projection-view of the antennal tip after confocal laser scanning microscopic investigation. The autofluorescence of the cuticle was used to identify the S. coelocapitula on the distal, ventral-lateral side of the antenna. The cuticular pit and cuticular-dense mushroom-like protrusion of the S. coelocapitulum is indicated by a higher autofluorescence signal (arrowheads). **(B)** Transmission light microscopy of the S. coelocapitulum. The mushroom-like protrusion and the cuticular pit below the antennal surface cause different light refraction. The circular structure with a central cuticular protrusion shows no extruding hair structure when focused through different levels (arrowheads). **(C,D)** Extracellular recording (bottom line) of the sensory neurons associated with the S. coelocapitulum. The neuronal signals of two different sensory neurons can be distinguished after spike sorting and template matching (upper line).

The S. coelocapitulum can also be identified under light microscopic and cLSM conditions. Using cLSM, the autofluorescence of the mushroom-like protrusion and the cuticular depression allows unambiguous identification (Figure 3A). The pit and the mushroom-like protrusion of the S. coelocapitulum cause characteristic light refraction (Figure 3B) that allows the identification of the mushroom-like protrusion. Under light microscopic conditions, as we used for extracellular recordings of the associated sensory neurons (Figure 3C), characteristic structures are visible. After spike sorting of the recordings, two distinct sensory neurons can be identified by template matching (Figure 3D).

### The TT-Neuron Encodes Transient Temperature

**Figure 4:**
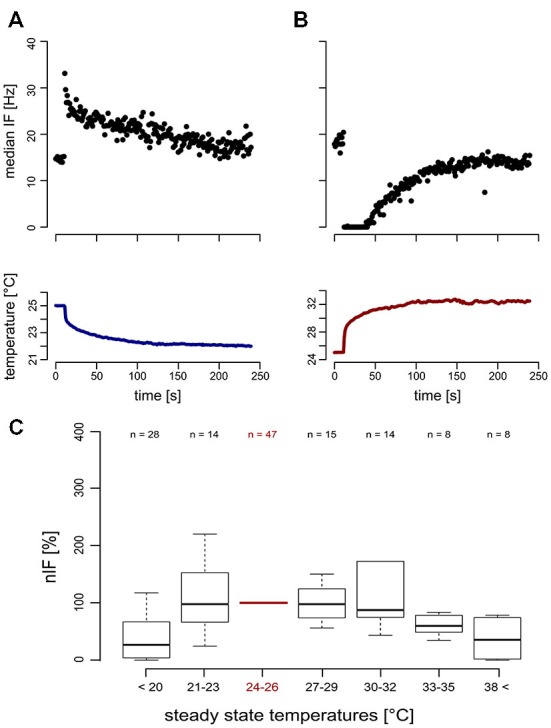
**Neuronal response (top) of transient temperature detectors (TT-neurons) and temperature stimulation (bottom). (A)** Phasic increase of neuronal activity in a TT-neuron during cold stimulation (blue). **(B)** Reduced neuronal activity of a second TT-neuron during heat stimulation (red). **(C)** The normalized instantaneous frequency (nIF) during steady state temperature conditions was stable in a temperature range from 21°C to 35°C, indicating a working range of TT-neurons of at least 14°C.

For temperature changes, the phasic response characterizes the TT-neurons as transient temperature detectors (flux-detectors; Figures 4A,B). Across different steady state temperature conditions, the resting activity of the TT-neurons was comparable. At the average steady state temperature of 24.98°C ± 0.53°C (range: 23.66–26.34°C, median = 25.04°C, *n* = 47) the resting activity was in a range between 10.66 Hz and 116.29 Hz (mean = 36.77 Hz, *SD* = 22.68 Hz, median = 32.03 Hz, *n* = 47). The resting activity at the starting temperature (around 25°C) was used to normalize the resting activity at different steady state temperatures in the range of 16–48°C (Figure 4C). Each neuron was measured in one or up to all six temperature categories that were presented randomly, and repeated measurements within one category were omitted. Since we obtained independent and paired data across the temperature categories, we did not conduct any statistical analysis. The median nIF of TT-neurons was very similar for steady state temperatures in the range between 21°C and 35°C, and at temperatures below 21°C and above 38°C the resting activity of the TT-neurons ceased. The latter temperatures may indicate the limits of the working range of the TT-neurons (Figure 4C). The adaptation property of the TT-neurons to distinct steady state temperatures does not allow the encoding of absolute temperature.

**Figure 5:**
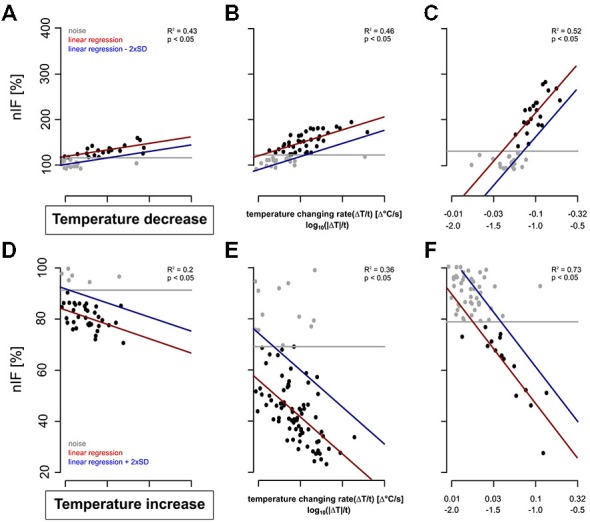
**Examples of dose-response curves (red) of six TT-neurons.** The neuronal activities of the cold-sensitive neurons 240 s after stimulus onset are represented in gray and black. The noise level of the resting activity is indicated by the gray line and that of the neuronal response during temperature stimulation in blue. **(A–C)**: Three TT-neurons stimulated with cold air stimulus with **(A)** low differential sensitivity and low detection threshold, **(B)** intermediate differential sensitivity and low detection threshold, and **(C)** high differential sensitivity and high detection threshold. **(D–F)**: Three TT-neurons stimulated with increasing temperature with **(D)** low differential sensitivity and low detection, **(E)** intermediate differential sensitivity and low detection threshold, and **(F)** high differential sensitivity and high detection threshold.

The TT-neurons of the S. coelocapitulum varied in their detection threshold and their differential sensitivities to temperature stimulation. For a total of 20 TT-neurons, we investigated the correlation between the temperature changing rate (stimulus intensity) and the phasic response of the neurons in a time-window of 240 s after stimulus onset. All the TT-neurons differ considerably in their response characteristics (Figure 5). However, irrespective of these differences, we calculated mean values for the detection threshold, the differential sensitivity and the resolving power during cooling (Figures 5A–C) and heating (Figures 5D–F), respectively.

The detection threshold of the recorded TT-neurons for a temperature decrease ranged from −0.003°C/s to −0.102°C/s (mean = 0.047°C/s, *SD* = 0.031°C/s, *n* = 20, Figures 5A–C). Neurons with a higher resting activity had a higher noise level, e.g., a higher detection threshold (Pearson’s correlation index for unpaired data: 0.80, *p*: <0.05, *n* = 20). During heating, the activity decreased in most cases (*n* = 16) to complete inactivity. However, in some TT-neurons (*n* = 4) the activity dropped to a nIF of about 20% and the activity level was maintained for several seconds. This period of reduced activity was used for calculating the dose-response curve of the TT-neurons for increasing temperature (Figures 5D–F). The detection threshold for a temperature increase ranged from 0.006°C/s to 0.124°C/s (mean = 0.029°C/s, *SD* = 0.029°C/s, median = 0.020°C/s, *n* = 15, Figures 5D–F). Based on our linear regressions, the differential sensitivity during cold stimulation ranged from 25.05% to 298.95% (mean = 105.58%, *SD* = 86.07%, median = 60.80%, *n* = 20) when stimulus intensity changed an order of magnitude (e.g., from 0.01–0.1°C/s). The differential sensitivity during temperature increase ranged from −11.42% to −89.38% (mean = −32.33%, *SD* = 18.24%, median = −27.26%, *n* = 15). The differential sensitivity (slope of linear regression) was not dependent on noise level (Pearson’s correlation index for unpaired data: −0.32, *p*: 0.17, *n* = 20).

The resolving power for temperature changes was stable across individual neurons. The resolving power of the single TT-neurons was calculated as a factor describing the difference in stimulus intensity needed to elicit a different nIF with a probability of 90%. For decreasing temperatures, the resolving power of TT-neurons ranged from 0.019–0.837 (mean = 0.372, *SD* = 0.260, median = 0.314, *n* = 20). For example, a TT-neuron excited at a temperature changing rate of −0.1°C/s will change its nIF significantly when stimulus intensity changes by 0.037°C/s (e.g., −0.063°C/s or −0.137°C/s). The resolving power of the TT-neurons during temperature increase was between 0.083 and 0.506 (mean = 0.275, *SD* = 0.133, median = 0.240, *n* = 15).

Since TT-neurons differed in their physiological properties, we asked whether resting activity of the neurons correlates with the detection threshold and the differential sensitivity. The detection threshold and the differential sensitivity did not correlate (Pearson’s correlation, *p* = 0.77, *n* = 20), thus TT-neurons seem to have a neuron specific combination of detection threshold and differential sensitivity. We further investigated the same parameters for temperature decreases after adaptation to higher and lower temperatures (adaptation-time: 500 s). This allowed us to address the question, if the physiological properties depend on steady state temperature conditions. We measured the neurons’ responses to temperature decrease starting at rearing temperature (25°C) and once more starting between 27°C and 38°C. The latter we classified as heat-adapted. We found no significant differences in physiological properties when TT-neurons were heat-adapted (Mann-Whitney-U-Test: *p* = 0.25 for detection threshold, *p* = 0.95 for differential sensitivity, *p* = 1 for resolving power, *n* = 8).

Additionally, the physiological properties for increasing temperature were investigated after adaptation to a temperature between 18°C and 23°C (cold-adapted). The detection threshold and the differential sensitivity of the TT-neurons were not significantly different under different ambient temperature conditions (*p* = 0.84 for detection threshold, *p* = 0.84 for resolving power, *n* = 6). Only the differential sensitivity showed a significant increase for cold-adapted TT-neurons (*p* = 0.03, *n* = 6).

### The ST-Neuron has a Narrow Working Range

**Figure 6:**
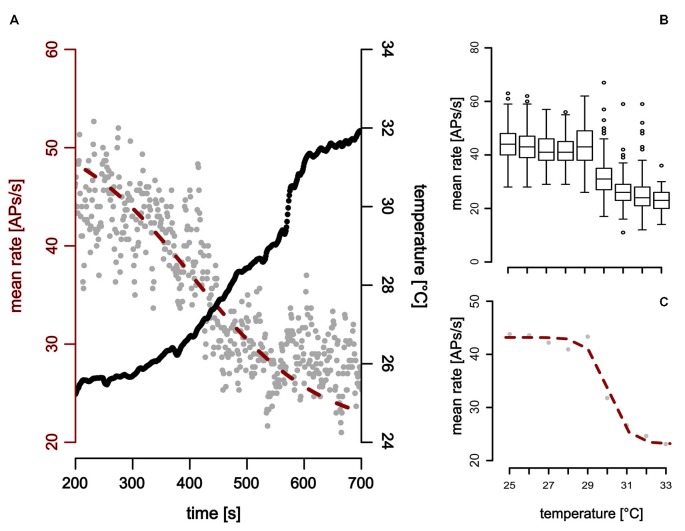
**Neuronal response of a ST detector (ST-neuron) to gradual temperature increase.** The neuronal response shows two distinct activity levels at the beginning and the end of the temperature stimulation. **(A,B)** The activity level at higher temperatures is characterized by a mean activity that does not change when temperature was increased further (Note that this is the case even at sudden temperature changes, e.g., around 600 s in **(A). (A,C)** A sigmoidal fit describes the activity of the neuron during temperature increase (red).

The second cold-sensitive neuron with smaller spike-amplitudes could be described when temperature was gradually increased (Figure 6). Increasing the temperature revealed a narrow working range in which the neurons are sensitive to temperature. No change in the neuronal activity was observed when temperature was further decreased below 25°C or increased above 32° (Figure 6).

**Figure 7:**
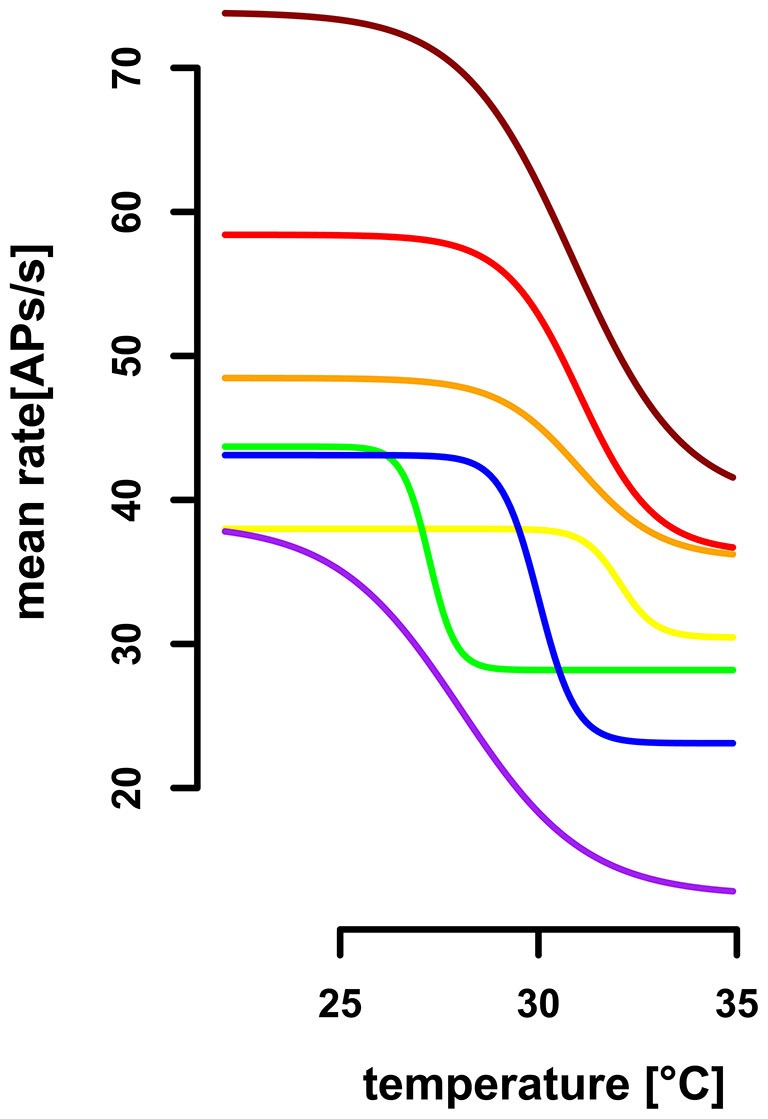
**Extrapolated dose-response curves for seven ST-neurons.** Sigmoidal regressions were calculated for seven ST-neurons (color-coded), based on their activity profile during gradual temperature increase. The working range of ST-neurons was between 26.87°C (*SD* = 2.51, range: 22.4°C–30.5°C) and 33.2°C (*SD* = 2.47, range: 28.4°C−35.9°C). The highest differential sensitivity (slopes at the turning points) range from 3.08AP/°C to 11.49AP/°C (*SD* = 3.29, mean: −6.48AP/°C).

In comparison to the TT-neurons, the ST-neurons were much more homogeneous, with an average resting activity of 50.0 APs/s (range: 35.4 APs/s–75.3 APs/s, *SD* = 14.8 APs/s, *n* = 7) at a temperature of 25°C (*SD* = 0.4°C, *n* = 7). Compared to the activity levels at 25°C, a significantly lower resting activity (mean = 29.5 APs/s, *SD* = 9.4 APs/s, *n* = 7) was found for temperatures above 32°C (mean = 33.3°C, *SD* = 1.0°C, *n* = 7; Mann–Whitney-U-Test for paired data: *p* < 0.004). The difference between the two levels of resting activity was on average 40.42% (*SD* = 15.92%, range: 16–69%, *n* = 7). We assessed the working range of the ST-neurons by fitting a sigmoidal curve to the activity data (Figure 6). The working range of ST-neurons was between 26.9°C (*SD* = 2.5°C, range: 22.4–30.5°C, *n* = 7) and 33.2°C (*SD* = 2.5°C, range: 28.4–35.9°C, *n* = 7; Figure 7).

We evaluated the differential sensitivity of the neurons to temperature increase by the slope of the sigmoidal fit at the turning point. The mean of highest sensitivity was −6.5 APs/°C (*SD* = 3. 3 APs/°C, range: 3.1 –11.5 APs/°C, *n* = 7, Figure 7), at an average temperature of 30.0°C (*SD* = 1.8°C, range: 27.2–32.0°C). We conclude that the neurons are insensitive to temperature changes below 26.9°C and above 33.2°C, but are sensitive to steady state temperatures in between. The resolving power was calculated for each ST-neuron, based on a linear regression in the range of 1°C around the turning point of the sigmoidal fit, respectively, and resulted in a range from 0.31°C to 2.90°C (mean = 1.58°C, *SD* = 0.99°C, *n* = 7). Thus, the ST-neurons differ greatly in their ability to code for different steady state temperatures.

## Discussion

The S. coelocapitulum in the carpenter ant *Camponotus rufipes* houses two cold-sensitive neurons that encode distinct information about the thermal environment. One of the sensory neurons is a flux-detector providing information about temperature changes over a wide working range (TT-neuron). The second cold-sensitive neuron has a narrow thermo-sensitive working range which covers the temperature range preferred by *C. rufipes* workers during brood care (Weidenmüller et al., 2009). TT-neurons and ST-neurons provide information about temperature transients and steady state temperature, respectively. We propose that the TT-neurons provide information suited to orient in inhomogeneous thermal environments, whereas the ST-neuron provides the ant information about preferable temperatures for e.g., brood depositing.

### Morphological Characteristics of the S. coelocapitulum

The S. coelocapitulum is a rarely described sensillum type, especially in ultrastructural and physiological terms. Although it can be found in many different insect species, earlier reports focused mainly on the external morphological characteristics and the location on the antenna in Hymenoptera (Yokohari et al., 1982; Yokohari, 1983; Agren and Hallberg, 1996; Ehmer and Gronenberg, 1997; Gnatzy and Jatho, 2006; Nakanishi et al., 2009; Ramirez-Esquivel et al., 2014; Ravaiano et al., 2014). S. coelocapitula have also been described in other insect families like Mantodea and Coleoptera as well (Giglio et al., 2008; Carle et al., 2014). Fine-structural investigations and physiological examinations of this inconspicuous sensillum are very limited (Yokohari, 1983; Tichy and Kallina, 2014). By combining SEM, light microscopy and sensory physiology, we present an approach for unambiguous identification and provide a comprehensive description of the S. coelocapitulum in ants.

The sensillum can be identified by its mushroom-like protrusion within a shallow depression of the antenna, and our findings of the outer morphology are very much comparable to descriptions of the S. coelocapitulum in other Hymenoptera (Dietz and Humphrey, 1971; Yokohari et al., 1982; Yokohari, 1983; Nakanishi et al., 2009). In *Apis mellifera*, the S. coelocapitulum houses a sensory triad of two hygro- and one thermo-sensitive neuron (Yokohari, 1983). The physiological properties of the two hygro-sensitive neurons, one moist- and one dry-sensitive neuron were described recently but the detailed investigation of the thermo-receptive neuron is still missing (Tichy and Kallina, 2014). The number of S. coelocapitula on the antenna of ants is low and they are localized in two distinct clusters, one at the most distal and one at the most proximal segment of the antenna (Nakanishi et al., 2009). Whether there are few, scattered S. coelocapitulum on the other segments of the flagellum, as described for *Camponotus japonicus* (Nakanishi et al., 2009) is unknown and was not investigated in this study. In beetles, a very comparable sensillum type is located at the tip of the flagellum. Its external morphology consists of a cuticular dome with a central, mushroom-like protrusion; however, it is named “S. campaniform” (Merivee et al., 1998, 1999, 2003; Must et al., 2006a,b).

The annotation in beetles is misleading, since campaniform sensilla are stimulated by mechanical forces and assigned to proprioception (Schmidt and Gnatzy, 1971; Spinola and Chapman, 1975; Zill and Moran, 1981). Additionally, the morphological characteristics of the “S. campaniform” in beetles are more similar to the S. coelocapitulum of the honey bee (Giglio et al., 2008; Di Giulio et al., 2012). The “S. campaniform” in Coleoptera house three sensory neurons, of which one is cold-sensitive (Merivee et al., 2003; Must et al., 2006a,b), therefore the sensillum has to be considered as being a S. coelocapitulum with coleopteran-specific morphological modifications, e.g., cuticular dome instead of a depression around the sensory peg. The fine-structure of the S. coelocapitulum in *C. rufipes* verifies the sensillum as a non-porous (np) sensillum, similar to the S. coelocapitulum in honey bees (Yokohari, 1983). The combined investigation by TEM and by FIB-FESEM resulted in a detailed analysis of the cuticular apparatus. The peg is attached within the antennal cuticle at an oblique orientation. The distal part of the pit is air-filled, and might insulate the sensory peg from the antennal lumen.

Thermo-sensitive neurons have not been described for single walled (sw) sensilla with porous walls and a combination of olfactory and thermo-sensitive neurons is only described for double walled (dw) sensilla with spoke channels (Schaller, 1978; Altner and Loftus, 1985). In some sensilla with thermo-receptive function, the outer dendritic segments of the thermo-sensitive neurons are lamellated, and this trait has been discussed as being indicative for thermo-sensitive neurons where increased membrane area at the dendrites may support increased sensitivity, an idea that has never been investigated specifically (Steinbrecht et al., 1989). Large membrane areas at the outer dendritic segments can also arise by multiple branches, and indeed this has been described for other thermo-sensitive neurons associated with S. coeloconica in ants (Ruchty et al., 2009). Based on our fine-structural investigations, we found no indication of lamellation or branching at the outer dendritic segment, however, we cannot rule out this possibility.

It remains an open question, why thermo-sensitive neurons with similar physiological properties are associated with very different sensilla across insects. One possible explanation is that morphological structures of thermo-receptive sensilla evolved to serve other functions than thermo-reception, e.g., olfaction or humidity-reception by other sensory neurons being present in the same sensillum. For example, olfactory neurons require sensilla with pores or spoke channels as described for the S. coeloconica in leaf-cutting ants (Ruchty et al., 2009). In the cockroach *Periplaneta americana*, two cold-receptive neurons are located in two different types of sensilla, encoding information about temperature changes and steady state temperature. One of the thermo-sensitive neurons is associated with a dw-sensillum that additionally contains olfactory neurons, encoding temperature changes comparable to the TT-neuron described in our study. The other one, encoding temperature changes and steady state temperatures, is located in a np-sensillum that additionally contains hygro-sensitive neurons (Nishikawa et al., 1992). Comparable to the latter case, we described a similar neuron (ST-neuron) also in a np-sensillum type, and it remains to be investigated whether and how the measurement of steady state temperature is improved in np sensilla.

The cryptic structure and the simple organization of the S. coelocapitulum are possibly advantageous for thermo-reception. A plausible selective pressure is on the low mass of the S. coelocapitula, leading to fast stimulus transduction by reducing thermal hysteresis. Np sensilla are always sw and previously sw-sensilla were considered as being basal to dw-sensilla (Altner and Prillinger, 1980; Keil, 1999). However, the expression of ancient IR-receptors in dw-sensilla coeloconica of *Drosophila* is challenging this hypothesis (Benton et al., 2009), and possibly dw-sensilla are basal with respect to sw-sensilla.

Irrespective of the origin of dw/sw-sensilla, the variety we find in other, more prominent and highly organized dw-sensilla with thermo-sensitive neurons seems to be the result of adaptations to neurons tuned to other sensory modalities than thermo-reception. Thermo-sensitive neurons remained in these sensilla throughout the evolution of sensilla types or were “recruited” at a later stage, and the latter might be true also for neurons/receptors for other sensory modalities.

### The Physiological Properties of the Thermo-Sensitive Neurons

Cold-sensitive neurons that respond to temperature changes with a phasic-tonic response dynamic were found in various insect species (Lacher, 1964; Loftus, 1968; Waldow, 1970; Tichy, 1979; Nishikawa et al., 1992; Merivee et al., 2003; Must et al., 2006a,b; Ruchty et al., 2009, 2010a,b). The phasic response of cold-sensitive neurons relates to a change in temperature (temperature changing rate), however, the resolving power of the different systems was rarely quantified (Loftus and Corbière-Tichané, 1981; Ameismeier and Loftus, 1988; Zopf et al., 2014). The tonic-phase of the response of phasic-tonic cold-sensitive neurons may change at prolonged steady state temperature conditions [*P. oblongopunctatus*, Must et al. (2006b); *A. mellifera*, Lacher (1964); *L. migratoria*, Ameismeier and Loftus (1988), Waldow (1970); *A. aegypti*, Davis and Sokolove (1975); *Platynus assimilis*, Must et al. (2006a) *Periplaneta americana*, Nishikawa et al. (1992)]. Such response properties led several author to conclude that this type of neuron may code for two parameters of thermal information: temperature changes and steady state temperatures. However, the classification of tonic and phasic responses in these systems is inconsistent, since even slowly adapting tonic responses are unsuited to code for steady state conditions. Cold-sensitive neurons that transiently code for temperature changes with same resting activities at different temperatures had been described for several insects *P. cupreus*, Must et al. (2006b); *P. Americana*, Loftus (1968), Nishikawa et al. (1992); *Anchomences dorsalis* and *Agonum muelleri*, Must et al. (2006a). The only cold-sensitive neuron described in detail in ants is associated with the S. coeloconicum in the leaf-cutting ant *Atta vollenweideri* (Ruchty et al., 2009, 2010a,b). The adaptation property increases the temperature range in which thermal changes can be detected, but information about steady state temperatures is not maintained, and the orientation of the pit and the peg of the sensory neurons implies a direction sensitivity of the sensory neurons used for orientation (Ruchty et al., 2009, 2010b). The sensitivity of these neurons was described by the *k*-value (exponent) of the Steven’s power function that allows comparison within and across modalities (Ruchty et al., 2010b). The mean *k*-value for the cold-sensitive neurons in *Atta vollenweideri* is 0.52 (range: 0.26–0.71, *n* = 24; Ruchty et al., 2010b)). The TT- neurons of *C. rufipes* change their activity for a temperature change of −0.1°C/s by a factor between 0.25 and 2.99. Thus, for small temperature changes, the *k*-values measured *in A. vollenweideri* correspond well to our measure of differential sensitivity in *C. rufipes*, and we find a similar or even higher sensitivity to temperature changes.

Surprisingly, the resolving power of individual TT-neurons is similar across neurons and treatments. Our analysis, including the detection threshold and the resolving power provides a detailed estimate of the sensitivity of this sensory system. For a given detection threshold of a TT-neuron (mean = −0.047°C/s) and an average resolving power of 0.372, the smallest noticeable difference of a stimulus is −0.017°C/s (negative temperature stimulation). For positive temperature stimulation with an average detection threshold of 0.029°C/s (resolving power: 0.275), the smallest noticeable difference is only 0.008°C/s.

In contrast to the S. coeloconicum in *A. vollenweideri*, the morphological characteristics of the S. coelocapitulum do not indicate direction sensitivity. Although the direction sensitivity in S. coelocapitulum remains an open question, the distal location of the S. coelocapitulum indicates that temperature differences in the environment can be detected during locomotion and scanning movements of the antennae. This would allow fast detection of temperature gradients, which might be used for orientation (Kleineidam et al., 2007).

The working range of the TT-neuron covers most of the temperature range *C. rufipes* will experience in their nests throughout the year (Weidenmüller et al., 2009). The low detection thresholds and the high sensitivities of the TT-neurons provide the ant with information about temperature changes at a high temporal resolution. The measured variability in detection thresholds of the TT-neurons may reflect the sensory equipment of an individual. In that case, the receptive range for thermo-sensation is broadened by diverse but overlapping dose-response curves, as described for many other sensory systems.

Alternatively, each of the TT-neurons we measured may represent one of several neurons within an individual, which all have similar sensitivities, resulting in a much smaller individual sensitivity range than the sensitivity range we measured across individuals. In that case, inter-individual differences in detection thresholds can explain differences in behavioral sensitivity, e.g., thresholds for behavioral responses to temperature changes that have been described for individuals with different thermal experience during development (Weidenmüller et al., 2009). We propose that the properties of such a thermo-sensory system may contribute to division of labor in social insects. As stated by the “response threshold models”, inter-individual variability in intrinsic behavioral response thresholds can cause variation in individual task preference (reviewed in Beshers and Fewell, 2001).

The other cold-sensitive neuron (ST-neuron) within the S. coelocapitulum responds to temperature only within a narrow working range. The narrow working range of the ST-neurons between 26.87°C and 33.2°C matches the previously described temperature range *C. rufipes* worker prefer for their brood (Weidenmüller et al., 2009). This leads us to hypothesize that this cold-sensitive ST-neuron may act as a thermal switch, triggering directly the temperature guided brood care behavior. Behavioral tests revealed a modulation of behavioral thresholds during brood care when pupae were reared at different steady state temperature conditions (Weidenmüller et al., 2009). Experience dependent modulation of the working range of ST-neurons is a possible proximate mechanism to adjust individual brood care behavior in *C. rufipes*. In this study, we show that different parameters of temperature information are extracted in parallel by two cold-sensitive neurons, both associated with the S. coelocapitulum. The resolving power of the TT-neurons for transient temperature is very high and in combination with the steady state temperature information of the ST-neurons the animals can assess the necessary information for fine-tuned thermal guided behavior.

## Conflict of Interest Statement

The authors declare that the research was conducted in the absence of any commercial or financial relationships that could be construed as a potential conflict of interest.
